# Circulating and tumor-associated caspase-4: a novel diagnostic and prognostic biomarker for non-small cell lung cancer

**DOI:** 10.18632/oncotarget.25049

**Published:** 2018-04-10

**Authors:** Michela Terlizzi, Chiara Colarusso, Ilaria De Rosa, Nicolina De Rosa, Pasquale Somma, Carlo Curcio, Alessandro Zamparelli Sanduzzi, Pietro Micheli, Antonio Molino, Antonello Saccomanno, Rosario Salvi, Rita P. Aquino, Aldo Pinto, Rosalinda Sorrentino

**Affiliations:** ^1^ Department of Pharmacy, University of Salerno, ImmunePharma S.r.l., Fisciano, SA, Italy; ^2^ Anatomy and Pathology Unit, Ospedale dei Colli, AORN, “Monaldi”, Naples, Italy; ^3^ Thoracic Surgery Unit, Ospedale dei Colli, AORN, “Monaldi”, Naples, Italy; ^4^ Department of Respiratory Medicine, Respiratory Division, University of Naples Federico II, Fisciano, SA, Italy; ^5^ PhD Program in Drug Discovery and Development, Department of Pharmacy, University of Salerno, Fisciano, SA, Italy

**Keywords:** non-small cell lung cancer, NSCLC, diagnosis, prognosis, biomarker

## Abstract

Late diagnosis limits therapeutic options and survival rate of non-small cell lung cancer (NSCLC) patients. Therefore the identification of biomarkers represents an emerging medical need.

A highly sensitive and specific test was developed to identify/quantify a novel/selective diagnostic biomarker for NSCLC patients, caspase-4. This test was validated by using i) plasma from 125 NSCLC patients and 79 healthy (non-pathological) subjects, ii) plasma from 139 smokers and iii) from 70 chronic-obstructive pulmonary disease (COPD) patients. Caspase-4 quantification was also assessed in the lung tumor mass of 98 paired NSCLC patients compared to 10 non-tumor lung tissues (i.e. tuberculosis).

Circulating caspase-4 was detected in both healthy and NSCLC patients; however at different range values: 2.603–3.372 ng/ml for NSCLC patients (95% CI) compared to 0.3994-0.6219 ng/ml for healthy subjects (95% CI). The sensitivity of the test ranged from 97.07% to 100%; the specificity was 88.1% with a positive predictive value of 92.54%, accuracy of 95.19% and AUC of 0.971. Smokers (95% CI, 0.3947–0.6197 ng/ml) and COPD patients (95% CI, 1.703–2.995 ng/ml) showed intermediate values of circulating caspase-4. Tissue levels of caspase-4 in the tumor mass showed that 72 (72.7%) out of 99 patients were positive. More importantly, higher levels (cut-off value = 0.307 ng/ml) of caspase-4 in the tumor mass were associated to reduced overall survival (median 0.92 years) compared to NSCLC patients with lower levels (median 3.02 years).

We report for the first time caspase-4 as a novel diagnostic and prognostic biomarker, opening new therapeutic perspectives for NSCLC patients.

## INTRODUCTION

Lung cancer is the third common cancer-related disease worldwide; however, it is the leading cause of cancer-related deaths and it counts more deaths than other solid tumors [1]. The number of new cases is expected to rise by about 70% over the next two decades; but more dramatically, over half of people with lung cancer die within one year from time of diagnosis with a survival rate less than 10% [1].

This dramatic condition is primarily due to the lack of early detection tools and to the recognition of the symptoms at the sole late stages, combined to poor pharmacological efficiency/benefit of the available therapeutic approaches. In the absence of a reliable screening tool, less than 15% of patients are diagnosed of early stage I lung cancer, and less than 15% of all patients survive for 5 years after the diagnosis. To date, more than 80% of patients are ineligible for surgical resection at the time of diagnosis, mostly because of the advanced stage of the cancer and for the poor general conditions at stage III-IV [2, 3]. One of the limitation to early diagnose lung cancer is that its initial development is radiologically occult.

Several screening have been developed so far to identify biomarkers to provide insights on diagnosis, prognosis and response to treatment [4]. Currently, the identification of biomarkers has been performed by using invasive and non-invasive procedures. In the context of lung cancer, the invasive procedures rely on histologic confirmation performed on biopsy specimens and on lung samples obtained by surgical resection, performed because of a high likelihood evidence of lung cancer diagnosis. Noninvasive procedures comprise screening of sputum cytology, which has been used with limited success [5, 6], and low-dose spiral computed tomography (CT) scanning, which limitations include high costs and the need for repeated scanning [5, 7].

Therefore, the research for biomarkers to be easily detected in serum by noninvasive procedures at an early stage of cancer has become an area of great interest for clinicians [8, 9].

Several biomarkers for lung cancer have been proposed to assist in the screening and early diagnosis of this type of cancer. However, limitations related to sensitivity and specificity of the (commercially) available diagnostic tests have been registered. Therefore, it is obvious that the non-invasive identification of selective, sensitive and specific biomarker/s represents the emerging medical need to avoid late diagnosis and ameliorate the personalized therapy with an ensuing higher survival rate and better lifestyle of lung cancer patients.

In this study we found that the circulating/tumor-associated caspase-4 is a novel diagnostic, predictive and prognostic biomarker for non-small cell lung cancer (NSCLC) patients.

## RESULTS

### Circulating caspase-4 is in the plasma of lung cancer (non-small cell lung cancer, NSCLC) patients

Blood from lung cancer patients, withdrawn before thoracic surgery, was tested for the presence of circulating caspase-4 by using a custom, not commercially available, ELISA kit developed by ImmunePharma S.r.l. (spin-off of the University of Salerno, Department of Pharmacy, DIFARMA, Italy).

Healthy subjects, included in this study, did not have any altered clinical parameter (i.e. CRP, cholesterol, etc.), non chronic respiratory diseases (i.e. non-COPD, non-Idiopathic Pulmonary Fibrosis (IPF), non-tuberculosis) and were non-smokers, in that they could be considered non-pathological, healthy (H).

**Figure 1:**
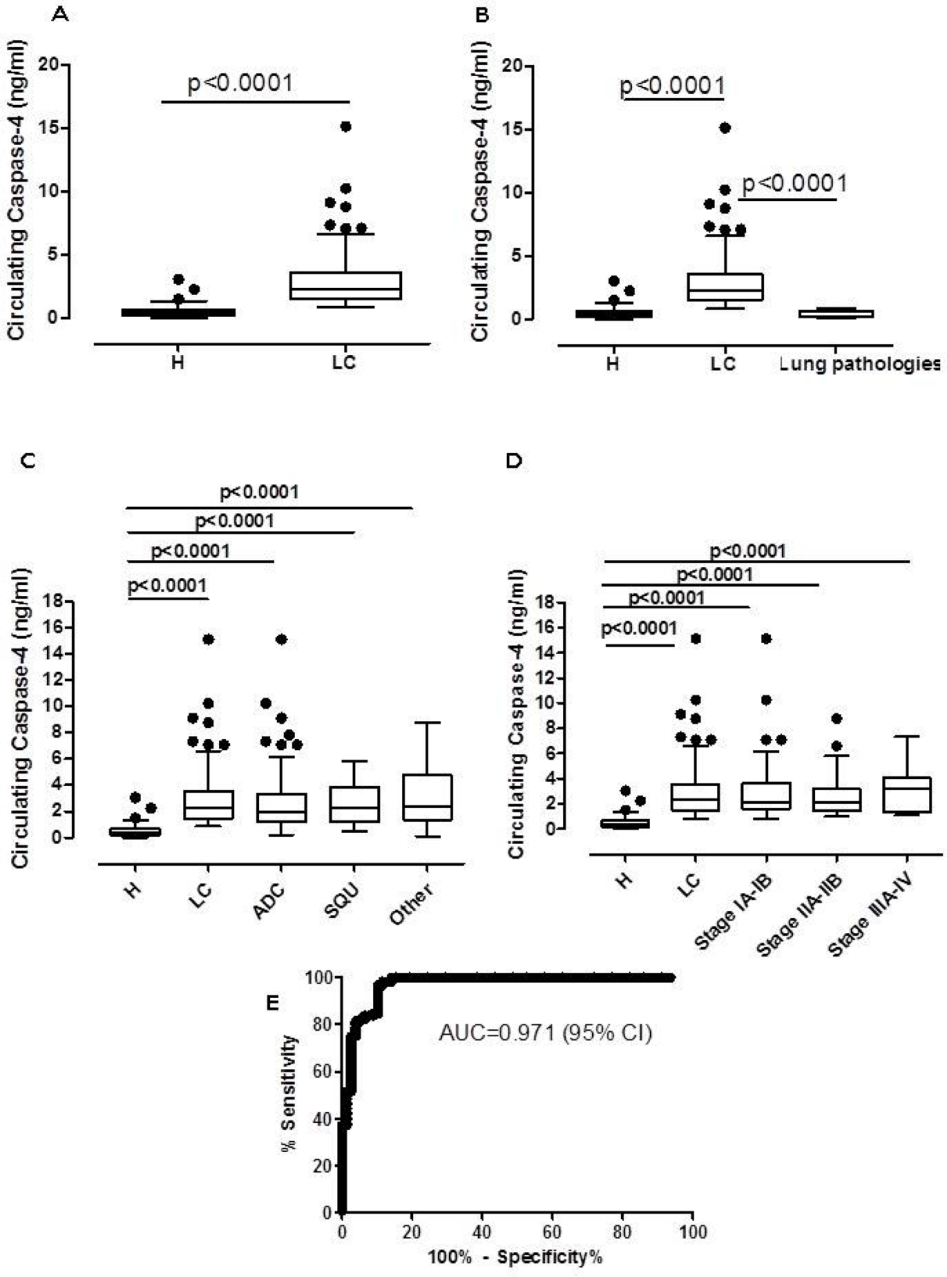
Circulating caspase-4 was in the plasma of NSCLC patients (**A**) Levels of caspase-4 were detected in the plasma of healthy (H, *n =* 79) and NSCLC (LC, *n =* 125) patients. H subjects were non-pathological. LC-derived blood was obtained before surgical resection of the tumor mass. Similarly, blood from patients who were not diagnosed of NSCLC or COPD (lung pathologies, *n =* 10) showed low levels of circulating caspase-4 (**B**). Levels of caspase-4 were analyzed according to the histotype (**C**) and stage (**D**) of NSCLC. (**E**) ROC analysis (H subjects vs LC) was performed to define the diagnostic features of caspase-4. Data are expressed as median ± interquartile range, showing outliers as dots. One Way ANOVA followed by Bonferroni’s post test was applied to (B, C and D). Mann Whitney test was performed for Figure A.

The levels of circulating caspase-4 were significantly higher in the plasma of lung cancer patients (LC; *n =* 125) than healthy non-smoker, non-pathological subjects (H; *n =* 79) (Figure 1A). In particular, plasma levels of the circulating caspase-4 were five-fold increased in lung cancer patients (95% CI, 2.603–3.372 ng/ml) compared to healthy subjects (95% CI, 0.3994–0.6219 ng/ml) (Figure 1A). To note, the circulating levels of caspase-4 were significantly low (*p <* 0.0001) in the plasma of patients (*n =* 10) who were diagnosed of chronic respiratory diseases (i.e. tuberculosis, emphysema) (here defined as lung pathologies), pathologies that were not related to COPD and/or lung cancer, compared to NSCLC patients (Figure 1B). These data ruled out the release of circulating caspase-4 in pulmonary inflammation.

To exclude the hypothesis that the observed differences could be related to the age, healthy subjects and lung cancer patients were stratified as ≤ or ≥ 60 years old. No statistical differences were observed in the levels of caspase-4 in the plasma of lung cancer patients who were ≤ or ≥ 60 years old (Table 1). Similarly, no differences were observed for healthy subjects who were ≤ or ≥ 60 years old (Table 1). To note, healthy subjects older than 60 still had lower levels of circulating caspase-4 compared to lung cancer patients older than 60 years old, ruling out age-related differences (Table 1).

**Table 1:** Levels of circulating caspase-4 in subjects older or younger than 60 years old

	Healthy (H)	Lung Cancer (LC)
**Age (years)**		
≥ 60	0.427 (0–1.245) *n =* 32	2.311 (0.854–15.13) *n =* 100
≤ 60	0.3971 (0–3.05) *n =* 47	2.28 (1.042–10.25) *n =* 25

Data are represented as median (median range).

In addition, the levels of circulating caspase-4 were also evaluated according to the histotype (Figure 1C) and stage (Figure 1D) of NSCLC. No statistical differences of circulating caspase-4 levels were observed in lung cancer patients according to the histotype (Figure 1C) and stage (Figure 1D). Moreover, no statistical differences were noted between males and females according to the histotype (Table 2) and stage (data not shown).

**Table 2:** Levels of circulating caspase-4 according to lung cancer histotype and gender

Lung Cancer	Adenocarcinoma	Squamous	Other
**Gender**			
Male	2.102 (0.725–15.13) *n =* 44	2.636 (1.062–5.825) *n =* 21	3.142 (1.195–8.773) *n =* 14
Female	2.198 (1.1–7.83) *n =* 26	2.944 (1.042–5.396) *n =* 8	2.029 (1226–5.825) *n =* 12

Data are represented as median (median range).

Based on these data, Receiver Operating Characteristic (ROC) curve was performed. Figure 1E shows that the area under the curve (AUC) was 0.971, implying an accuracy of the test that was higher than 90%, a value above which a biomarker can be defined as an excellent diagnostic biomarker. Moreover, we calculated a cut-off value according to the Youden’s index [10] which coincided with 0.506 ng/ml, that also represented the mean value of the circulating caspase-4 at the lower (0.3994 ng/ml) and upper (0.6219 ng/ml) 95% confidence interval (CI). According to the chosen cut-off, we could calculate false and true positive, and true and false negative values. In particular, we had: i. False negative (<0.506 ng/ml) = 0; ii. False positive (>0.506 ng/ml) = 10; iii. True negative (<0.506 ng/ml) = 69; iv. True positive (>0.506 ng/ml) = 125. According to MedCalc Software (https://www.medcalc.org/calc/diagnostic_test.php), the diagnostic test had the following features: Sensitivity = 100% (95% CI = 97.07% to 100%); Specificity = 88.10% (95% CI = 79.19% to 94.14%); Positive Likelihood ratio = 7.9; Negative Likelihood ratio = 0; Positive predictive value = 92.54%; Negative Predictive value = 100%; Accuracy = 95.19% (95% CI = 91.3% to 97.7%).

Based on these data, the circulating caspase-4 can be proposed as an excellent diagnostic biomarker for NSCLC.

### Circulating caspase-4 is selective for lung cancer (non-small cell lung cancer, NSCLC) patients

**Figure 2:**
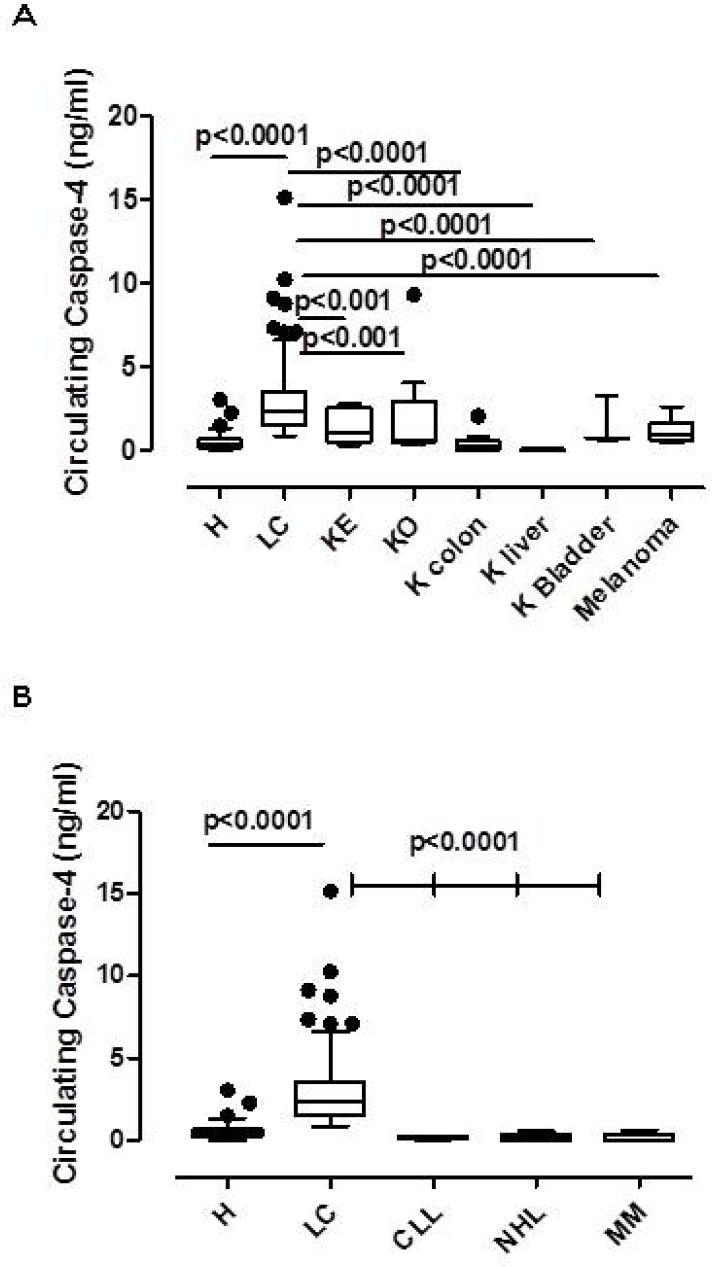
Circulating caspase-4 was solely present in the plasma of NSCLC patients Levels of circulating caspase-4 in NSCLC were compared to other solid (**A**) (KE, endometrial cancer, *n =* 12; KO, ovarian carcinoma, *n =* 12; K colon, colon carcinoma, *n =* 16; K liver, liver carcinoma, *n =* 5, K bladder, bladder carcinoma, *n =* 6; melanoma, *n =* 9) and liquid (**B**) (chronic lymphocytic leukaemia, CLL, *n =* 12; non-Hodgkin lymphoma, NHL, *n =* 12; multiple myeloma, MM, *n =* 12). Data are expressed as median ± interquartile range, showing outliers as dots. One Way ANOVA followed by Bonferroni’s post test.

In order to evaluate whether the circulating caspase-4 was present in other types of cancer, we tested its values in the blood obtained from patients with some solid and liquid tumors. In particular, the levels of the circulating caspase-4 were significantly lower in the plasma of endometrial carcinoma (KE, *n =* 12), ovarian carcinoma (KO, *n =* 12), colon carcinoma (K colon, *n =* 16), liver carcinoma (K liver, *n =* 5), bladder carcinoma (K bladder, *n =* 6) and melanoma (*n =* 9) (Figure 2A). Similarly, liquid tumors were evaluated. We found that the circulating caspase-4 was significantly lower in chronic lymphocytic leukaemia (CLL, *n =* 12), non-Hodgkin lymphoma (NHL, *n =* 12) and multiple myeloma (MM, *n =* 12) (Figure 2B).

These data imply that the circulating caspase-4 is selectively present in the blood of the sole NSCLC patients compared to the other cancer patients we evaluated.

### Circulating caspase-4 is detectable in the plasma of smokers and COPD patients

**Figure 3:**
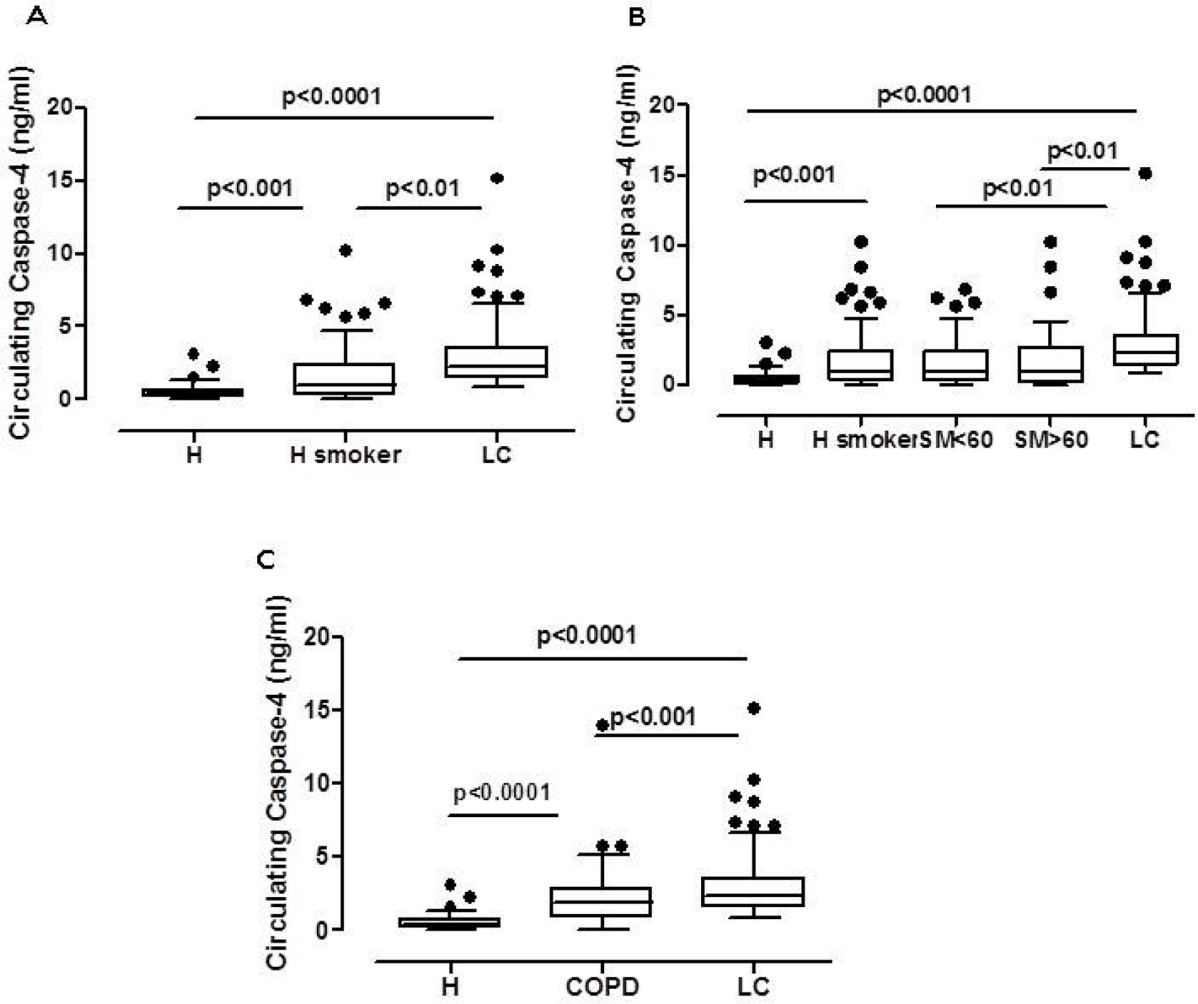
Circulating caspase-4 was detectable in the plasma of smokers and COPD patients Levels of circulating caspase-4 was analyzed in smokers, *n =* 139, (**A**) according to the age (**B**) (smokers, SM < 60 and SM > 60 years old). Similarly, levels of caspase-4 were analyzed in the plasma of COPD patients, *n =* 70 (**C**). Data are expressed as median ± interquartile range, showing outliers as dots. One Way ANOVA followed by Bonferroni’s post test.

In NSCLC patient cohort, 87.2% of lung cancer patients were smokers vs 12.8% of non-smokers (Table 3). Although, we did not find statistical differences in the circulating caspase-4 values between smoker vs non-smoker lung cancer patients (Table 3), it is widely known that smoking represents the highest risk factor for lung cancer [11]. Therefore, in order to evaluate the predictive power of the circulating caspase-4, the blood of smoking healthy subjects (>15 cigarettes/day) (*n =* 139) was tested. We found that the circulating caspase-4 was three-fold increased (95% CI, 1.331–1.94 ng/ml) compared to healthy subjects (95% CI, 0.3947–0.6197 ng/ml), although at lower levels than in lung cancer patients (95% CI, 2.603–3.372 ng/ml) (Figure 3A). Moreover, we did not find statistical differences between circulating levels of caspase-4 in subjects who were younger (SM < 60, *n =* 100) or older (SM > 60, *n =* 39) than 60 years old (Figure 3B), further highlighting that the levels of the circulating caspase-4 were not age-related as previously reported (Table 1).

**Table 3:** Levels of circulating caspase-4 according to smoking status

	Number patients	Lung cancer
Smoker	109 (87.2%)	2.279 (0.854–15.13)
Non-smoker	16 (12.8%)	2.764 (1.103–6.600)

Data are represented as median (median range).

Because COPD represents a chronic respiratory disease majorly caused by smoking, and that could be a risk factor for lung cancer [12], the levels of the circulating caspase-4 in the blood of COPD patients (*n =* 70) were also evaluated. We found that the levels of the circulating caspase-4 in COPD patients was three times higher (95% CI, 1.703–2.995 ng/ml) than healthy subjects (Figure 3C), although these levels were still lower than those observed in lung cancer patients (Figure 3C). Similarly to smokers, we did not have follow-up information for COPD patients.

### Tissue levels of caspase-4 are prognostic for lung cancer patients

In order to evaluate the tissue levels of the caspase-4 (defined as tumor-associated caspase-4), we used the ELISA kit developed by ImmunePharma S.r.l.

**Figure 4:**
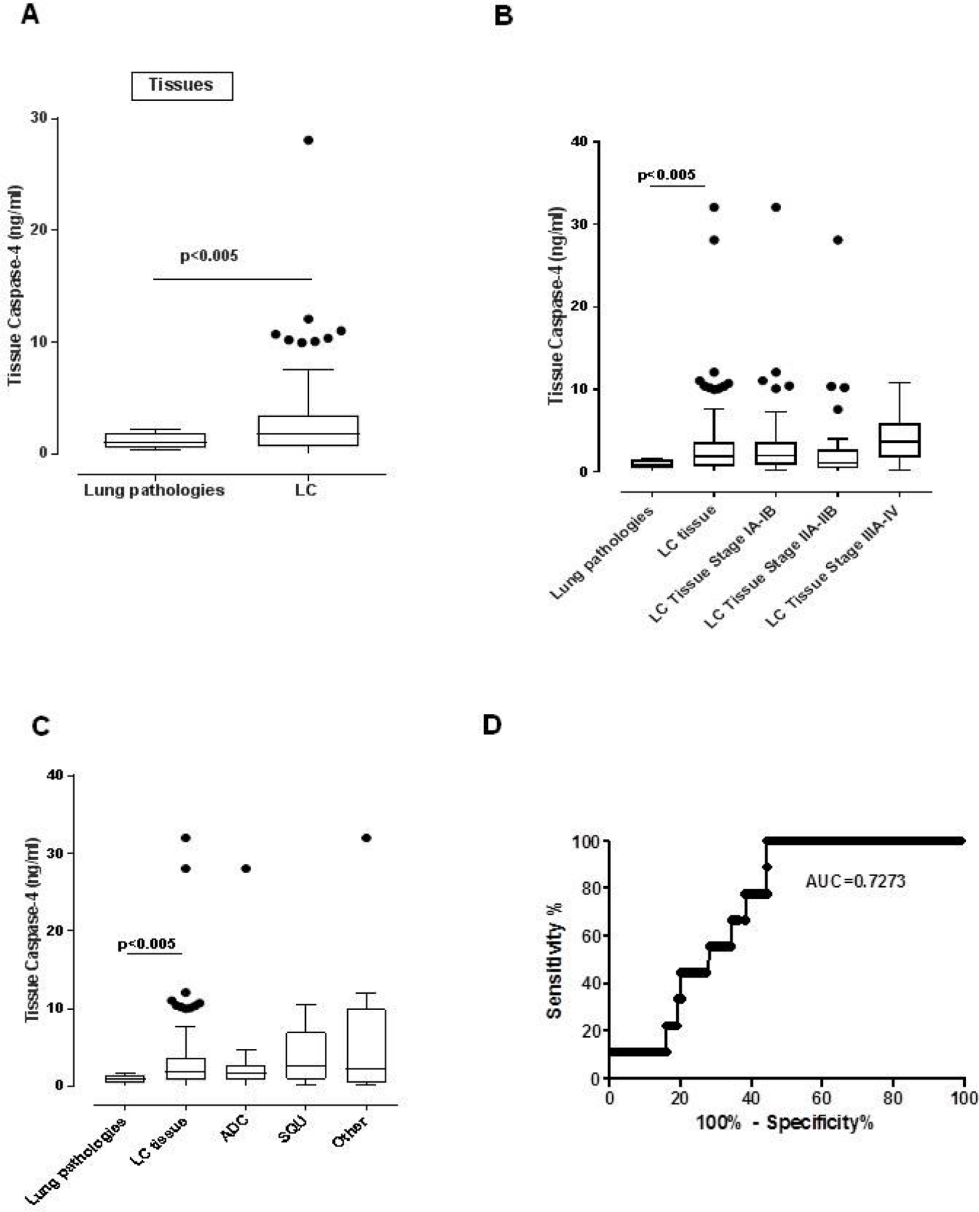
Tissue caspase-4 was detectable in the tumor mass of NSCLC patients (**A**) Levels of caspase-4 were detected in the tumor mass of NSCLC (LC, *n =* 99) patients compared to lung tissues obtained from non-cancer and non-COPD patients (i.e. tubercolosis) (lung pathologies, *n =* 10). Levels of tumor-associated caspase-4 were analyzed according to the stage (**B**) and histotype (**C**) (**D**) ROC analysis (lung pathologies vs LC) was performed to define the diagnostic features of caspase-4 starting from tissue samples. Data are expressed as median ± interquartile range, showing outliers as dots. One Way ANOVA followed by Bonferroni’s post test was applied.

We found that the levels of caspase-4 were significantly increased in NSCLC tissues compared to the lung tissues obtained from non-COPD, non-lung cancer (ie. Tuberculosis) patients (Figure 4A). In particular, we found that 72 (72.7%) out of 99 patients (Tables 4 and 5) were positive to caspase-4, according to a cut-off value of 0.377 ng/ml, which represented the mean value of the 95% CI of non-COPD, non-lung cancer tissues. Similarly to plasma levels we did not find statistical differences among the stages (Figure 4B) and histotypes (Figure 4C). Moreover, ROC curve showed that the AUC was 0.7273, implying that, although at lower levels than the plasma, tissue levels of caspase-4 tested by means of ELISA could represent a novel diagnostic tissue biomarker for NSCLC patients (Figure 4D).

**Table 4:** Positive vs negative staining of tissue caspase-4 according to the stage (IA-IB, IIA-IIB, IIA-IV)

		CASP4	
		Negative	Positive
**Stage**	IA-IB	12	41
	IIA-IIB	12	21
	IIIA-IV	3	10
	**Total**	**27**	**72**

**Table 5:** Positive vs negative staining of tissue caspase-4 according to the histotype (ADC = adenocarcinoma, SQU = squamous, other carcinoma)

		CASP4	
		Negative	Positive
**Histotype**	ADC	17	38
	SQU	4	18
	Other	6	16
	**Total**	**27**	**72**

**Figure 5:**
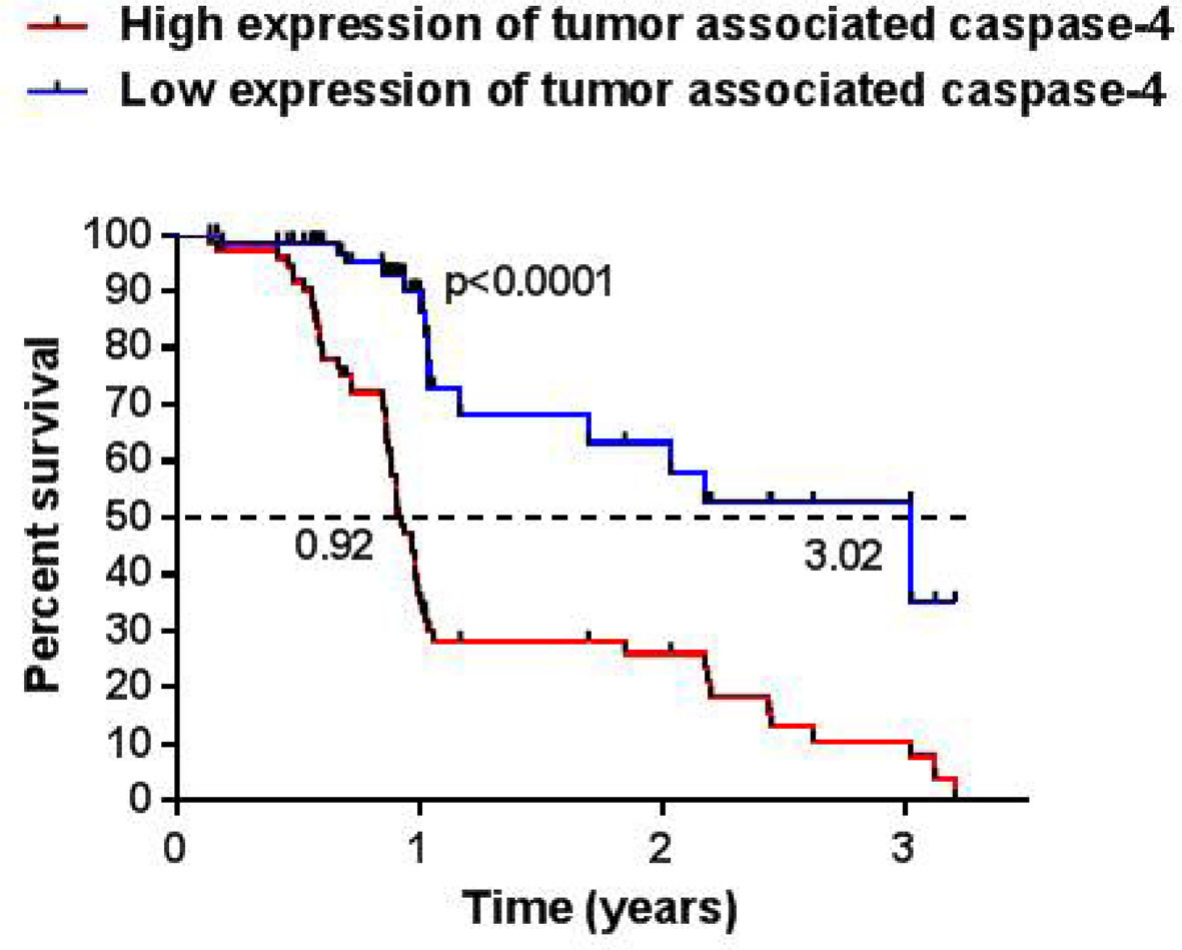
NSCLC patients with higher levels of caspase-4 in the tumor mass showed lower survival rate Tissue levels of caspase-4 were correlated to the available survival rate of NSCLC patients (*n =* 73). In particular, 59 NSCLC patients (80.8%) showed levels of tumor-associated caspase-4 higher than the cut-off (0.377 ng/ml); 14 NSCLC patients (19.2%) showed lower levels of caspase-4 than the chosen cut-off. Median survival of patients with high expression (red line) was 0.92 years vs median survival of patients with lower expression (blue line), 3.02 years. Log-rank Mantel-Cox and Wilcoxon test were performed to statistically analyze the survival rate between the two groups.

Moreover, in order to understand the prognostic value of the tumor-associated caspase-4, we analyzed the overall survival rate of NSCLC patients. The levels of tumor-associated caspase-4 were defined as low (<0.377 ng/ml) or high (>0.377 ng/ml) according to the cut-off value that derived from the mean of the tissue levels of caspase-4 from non-COPD, non-lung cancer patients (Figure 4A). High levels of caspase-4 (red line, Figure 5) were associated to lower survival rate compared to NSCLC patients who had lower levels of caspase-4 (blue line, Figure 5). Very interestingly, the median survival was of 0.925 months for NSCLC patients who had higher levels of tissue caspase-4 (>0.377 ng/ml). In contrast, NSCLC patients who had lower levels of caspase-4 (<0.377 ng/ml) in the tumor mass had longer median survival (3.02 years), which was three-fold higher compared to patients who had higher levels of caspase-4 (0.92 years). In particular, 80.8% of NSCLC patients (*n =* 58) had higher expression of caspase-4 compared to the patients with lower expression (19.2%, *n =* 14). According to the Log-rank Mantel-Cox and Gehan-Breslow-Wilcoxon test, the survival rate of NSCLC patients who had higher expression of tumor-associated caspase-4 was significantly lower (*p <* 0.0001) than those who had lower levels (Table 6).

**Table 6:** Statistical analysis for survival rate of NSCLC according to high (>0.377 ng/ml) and low (<0.377 ng/ml) expression of tumor-associated caspase-4

**Comparison of Survival Curves**	
Log-rank (Mantel-Cox) Test	
Chi square	27.84
df	1
*P* value	*P* < 0.0001
*P* value summary	^***^
Are the survival curves sig different?	Yes
Gehan-Breslow-Wilcoxon Test	
Chi square	27.76
df	1
*P* value	*P* < 0.0001
*P* value summary	^***^
Are the survival curves sig different?	Yes
Median survival	
>0.377	0.9250
<0.377	3.028
Ratio	0.3055
95% CI of ratio	–0.2529 to 0.8639
Hazard Ratio	
Ratio	3.446
95% CI of ratio	2.176 to 5.456

This table was obtained by using GraphPad Prism software 7.04.

Taken altogether, these data imply that the circulating, tumor-associated caspase-4 represents a novel selective diagnostic and prognostic biomarker.

## DISCUSSION

The identification of biomarkers for lung cancer by using non-invasive procedures has become an area of great interest in clinic. Currently, solely invasive procedures are reliable to define the high likelihood lung cancer diagnosis, implying that patients need to undergo thoracic surgery before a definite diagnosis, and even worse it has to be considered that not all patients that discover to be lung cancer patients are operable according to the stage and clinical status [13].

Our study demonstrates that circulating caspase-4 represents a novel, highly selective and sensitive diagnostic biomarker for NSCLC patients. We found that the AUC of the circulating caspase-4 is 0.971 with a high accuracy (95%) of diagnosing NSCLC before undergoing surgical procedures. Importantly, the diagnostic test has a very important feature in that it has high sensitivity and positive predictive value to detect lung cancer, implying very low false negatives, which nowadays represent the real limitation for the diagnostic tools available for NSCLC. However, the specificity (true negative vs false positive) was lower (88.1%) due to the presence of false positives, of whom we do not have a follow-up, implying that this value could be different if the subjects were followed-up in a time-dependent manner.

According to clinicaltrials.gov and recent published literature [4, 14–17], several biomarkers have been proposed for lung cancer diagnosis. In particular: 1. 15-gene mRNA signature; 2. serum NGAL, MMP-9, NGAL/MMP-9 complex, and soluble E-cadherin; 3. blood biomarkers related to i) hypoxia: Osteopontin (OPN), carbonic anhydrase IX (CA-9), and lactate dehydrogenase (LDH); ii) inflammation - interleukin 6 (IL-6), IL-8, and C-reactive protein (CRP), and α-2-macroglobulin (α-2M); and iii) tumor load: Carcinoembryonic antigen (CEA) and cytokeratin fragment (CYFRA 21-1); 4. blood levels of DCAMLK-1LK-1; 5. serum neuron specific enolase (NSE); 6. urine IGFBP-1, sIL-1Ra, CEACAM-1; 7. circulating microRNAs (miRNAs) such as let-7i-3p and miR-154-5p recognized as potential biomarkers of smoking-related lung cancer; 8. cell-free circulating tumor DNA (ctDNA); 9. gene mutations for K-RAS and Epidermal Growth Factor Receptor (EGFR) and Anaplastic Lymphoma Kinase (ALK); 10. Additional biomarkers currently being studied for application in cancer treatment include the PIK3CA, HER2, BRAF, ROS, RET, NRAS, MET, and MEK1. Nevertheless, noninvasive, circulating biomarkers so far under investigation and with potential clinical application are represented by CYFRA, NSE, CEA, CA 19.9, CA125 and circulating miRNA.

To note, the levels of sensitivity superior to 85% (value above which a biomarker could be considered as very good/excellent) are reached only after the combination of more biomarkers (CYFRA+NSE+CEA+CA125+CA19.9+ferritin), supporting the fact that each single biomarker has very low features of picking true positives. Therefore the combination of these available biomarkers has very low probability of diagnosing NSCLC in an accurate and efficient manner [18]. In addition, all the above biomarkers are common to other malignancies [18, 19], implying a non-selective presence, which could represent a major pitfall in case the patients undergo clinical evaluation for unknown unhealthy status. Another recently developed diagnostic/predictive test for eight common tumors, including NSCLC, is CancerSeek [20], based on multi-analyte blood non-invasive test. In the latter paper, NSCLC patients (*n =* 104) were screened according to the levels of the well-known gene mutations, such as K-Ras, p53, etc, versus protein levels, such as CEA, CA-125, CA19-9, osteopontin, etc. [20]. To our opinion, one great limitation of this test for NSCLC are the false negative values: 43 patients diagnosed of lung cancer were not positive to the multi-analyte blood test, implying lower sensitivity of the diagnostic multi-analyte test for NSCLC. Instead, our diagnostic tool proved that, although the false positive values (*n =* 10 out of 79 healthy subjects), patients clinically diagnosed of NSCLC were all positive to the screening, with no false negative results. False negatives represent the real limitation for a diagnostic biomarker in that a subject will show symptoms and obtain the final diagnosis at a late stage, limiting both surgery and therapeutic procedures. Moreover, in our study, circulating caspase-4 was solely detected at high levels (above the cut-off, 0.506 ng/ml) in NSCLC patients compared to some solid and liquid cancer patients, implying high selectivity of the test. Noteworthy, our diagnostic tool compared to the above described has higher levels of sensitivity, specificity and accuracy, with a positive likelihood of 7.9 and positive predictive value of 92.54. These results imply that a patient undergoing several screens to define a mass identified in the lung first by radiography and then by CT scan, could be easily identified at an early stage by evaluating the circulating levels of caspase-4.

Other non-invasive procedures comprise screening of sputum cytology, which has been used with limited success [5, 6], and low-dose spiral computed tomography (CT) scanning, which limitations include high costs and the need for repeated scanning [5, 7]. In the latter case, low-dose CT scan has been associated to the detection of around 21 miRNA to increase the sensitivity of the diagnostic test [21]. The presence of specific miRNA is correlated to lung cancer diagnosis [22], however, it still remains to understand whether the presence or not of one these miRNAs could change sensitivity and specificity of the diagnostic test.

Another emerging area of interest is represented by the analyses of the exhaled metabolites by means of mass spectrometry [23]. In this case, it is likely that daily status, food intake, lifestyle (ie. alcohol usage) could alter the levels of metabolites that might be non-specific for lung malignancy. In contrast, we found that besides the circulating, tissue levels of caspase-4 could represent a novel manner to diagnose lung cancer, although the ROC curve of the ELISA test on tissues was not as high as in the plasma. Nevertheless, we found that patients that were positive (high expression, >0.377 ng/ml) to caspase-4 had lower survival rate than negative (low expression, <0.377 ng/ml) patients. Median survival rate of patients with higher expression of caspase-4 in the tumor mass was three-fold decreased than patients with lower expression (0.92 vs 3.03 years) (Figure 5 and Table 6), implying that the levels of this protein in the tumor mass could represent not only a diagnostic tool but also a prognostic biomarker, opening new therapeutic perspective for NSCLC patients.

Another important issue to be pointed out is the higher presence of caspase-4 in the blood of smokers and COPD patients. It is well known that smoking represents the highest risk factor for lung cancer [11]. In our study we found that 70% of smokers and COPD patients were positive to the circulating caspase-4. Although we do not have a follow-up in the likelihood evidence of lung cancer for these subjects and patients, it is likely that the higher levels of the circulating caspase-4 compared to healthy subjects are predictive for a high risk of lung malignancy. It has to be noted though, that as already published, the environmental pollution together smoking is another risk factor for lung cancer. Indeed, we found that smokers- and COPD-derived PBMCs treated with air pollutants [24, 25], such as particulate matter (PM) of ultrafine sizes, were able to release caspase-4, index of higher risk for lung cancer establishment. A great limitation to this study is the absence of follow-up information about COPD and healthy smokers, in order to better understand and associate the levels of the circulating caspase-4 to the establishment of a tumor mass, avoiding late diagnosis.

In conclusion, in this study we propose the circulating and tumor-associated caspase-4 as a diagnostic biomarker for NSCLC that can be detected in both blood (in a non-invasive manner) and tissues with high sensitivity/true positive values compared to what so far described in the literature. It is more specific than currently available biomarkers (i.e. CA-9, CA-12.2, CEA), common to many non-neoplastic pathologies, and very importantly, it is related to the identification of a protein rather than DNA/RNA signature. In addition, tumor-associated caspase-4 has a prognostic value, opening new therapeutic perspectives for NSCLC patients.

## MATERIALS AND METHODS

### Human samples

The patients in this study were diagnosed with operable NSCLC (stage IA-IB, *n =* 79; Stage IIA-IIB, *n =* 34; Stage IIIA-IV, *n =* 12), and underwent surgical resection at Ospedale dei Colli, AORN, Monaldi, Naples, Italy, during the period 2014–2017. Clinical data were obtained from questionnaires and histology reports from the Pathological Anatomy Unit of the hospital. The project was approved by the institutional review board and by the Ethical Committee (approval number for lung cancer patients 1254/2014). Similarly, COPD-derived samples (only blood) were obtained after the approval of the institutional review board and Ethical Committee (approval number 11/2017). Blood from healthy non-smokers and smoker subjects were obtained by the same Unit at Ospedale dei Colli, AORN, Monaldi, Naples, Italy, when possible, and by “casa di Cura La Quiete”, Salerno, Italy, in collaboration (approval, 19.12.2016) with Immune Pharma S.r.l. Blood from healthy (smoker and non-smoker) subjects, COPD, lung cancer patients was collected after oral and written information provided by the MDs, and signed a written consent form before entering the project. EDTA-blood was obtained prior to surgery, in case of lung cancer patients.

Healthy (smoker and non-smoker) subjects were 40 ± 10 (mean ± S.E.M) years of age; COPD and lung cancer patients were 60 ± 10 (mean ± S.E.M) years of age. COPD and lung cancer patients were smokers or former smokers, whereas healthy subjects were divided in Smokers and Non-smokers.

Blood was withdrawn and collected and used within 24 hours.

### ELISA

The presence of the soluble caspase-4 was detected by means of ELISA, one of the technology patented by ImmunePharma S.r.l. (RM2014A000080 and PCT/IB2015/051262) (Department of Pharmacy, University of Salerno, Italy). The ELISA kit was based on custom antibodies, projected by the ImmunePharma S.r.l., and that are not currently commercially available. The test was performed on plasma and collagenase I (1 U/ml) digested lung tissues.

### Statistical analysis

Results are expressed as median ± interquartile range. One Way ANOVA, followed by Bonferroni’s post test, and/or Mann–Whitney test were used where appropriate. Percent survival was estimated by means of Kaplan–Meier method and compared with a non-parametric log-rank test. Percent survival was calculated from the time of surgical resection. The survival rate was calculated for 73 patients whose tissue-derived biological samples could be tested by means of ImmunePharma’s ELISA kit. *p* values less than 0.05 were considered significant.
