# Correction: Circulating and tumor-associated caspase-4: a novel diagnostic and prognostic biomarker for non-small cell lung cancer

**DOI:** 10.18632/oncotarget.25751

**Published:** 2018-06-29

**Authors:** Michela Terlizzi, Chiara Colarusso, Ilaria De Rosa, Nicolina De Rosa, Pasquale Somma, Carlo Curcio, Alessandro Sanduzzi, Pietro Micheli, Antonio Molino, Antonello Saccomanno, Rosario Salvi, Rita P. Aquino, Aldo Pinto, Rosalinda Sorrentino

**Affiliations:** ^1^ Department of Pharmacy, University of Salerno, ImmunePharma S.r.l., Fisciano, SA, Italy; ^2^ Anatomy and Pathology Unit, Ospedale dei Colli, AORN, “Monaldi”, Naples, Italy; ^3^ Thoracic Surgery Unit, Ospedale dei Colli, AORN, “Monaldi”, Naples, Italy; ^4^ Department of Respiratory Medicine, Respiratory Division, University of Naples Federico II, Fisciano, SA, Italy; ^5^ PhD Program in Drug Discovery and Development, Department of Pharmacy, University of Salerno, Fisciano, SA, Italy

**This article has been corrected:** The corrected author name is given below:

**Alessandro Sanduzzi**

Original article: Oncotarget. 2018; 9:19356-19367. https://doi.org/10.18632/oncotarget.25049

